# Pregnancy Weight Gain and Childhood Body Weight: A Within-Family Comparison

**DOI:** 10.1371/journal.pmed.1001521

**Published:** 2013-10-01

**Authors:** David S. Ludwig, Heather L. Rouse, Janet Currie

**Affiliations:** 1 New Balance Foundation Obesity Prevention Center, Boston Children's Hospital, Boston, Massachusetts, United States of America; 2 Arkansas Center for Health Improvement, University of Arkansas for Medical Sciences, Little Rock, Arkansas, United States of America; 3 Center for Health and Wellbeing, Princeton University, Princeton, New Jersey, United States of America; London School of Hygiene & Tropical Medicine, United Kingdom

## Abstract

**Background:**

Excessive pregnancy weight gain is associated with obesity in the offspring, but this relationship may be confounded by genetic and other shared influences. We aimed to examine the association of pregnancy weight gain with body mass index (BMI) in the offspring, using a within-family design to minimize confounding.

**Methods and Findings:**

In this population-based cohort study, we matched records of all live births in Arkansas with state-mandated data on childhood BMI collected in public schools (from August 18, 2003 to June 2, 2011). The cohort included 42,133 women who had more than one singleton pregnancy and their 91,045 offspring. We examined how differences in weight gain that occurred during two or more pregnancies for each woman predicted her children's BMI and odds ratio (OR) of being overweight or obese (BMI≥85th percentile) at a mean age of 11.9 years, using a within-family design. For every additional kg of pregnancy weight gain, childhood BMI increased by 0.0220 (95% CI 0.0134–0.0306, *p*<0.0001) and the OR of overweight/obesity increased by 1.007 (CI 1.003–1.012, *p* = 0.0008). Variations in pregnancy weight gain accounted for a 0.43 kg/m^2^ difference in childhood BMI. After adjustment for birth weight, the association of pregnancy weight gain with childhood BMI was attenuated but remained statistically significant (0.0143 kg/m^2^ per kg of pregnancy weight gain, CI 0.0057–0.0229, *p* = 0.0007).

**Conclusions:**

High pregnancy weight gain is associated with increased body weight of the offspring in childhood, and this effect is only partially mediated through higher birth weight. Translation of these findings to public health obesity prevention requires additional study.

*Please see later in the article for the Editors' Summary*

## Abstract 2

David Ludwig and colleagues examine the within-family relationship between pregnancy weight gain and the offspring's childhood weight gain, thereby reducing the influence of genes and environment.

*Please see later in the article for the Editors' Summary*

## Editors' Summary

**Background:**

Childhood obesity has become a worldwide epidemic. For example, in the United States, the number of obese children has more than doubled in the past 30 years. 7% of American children aged 6–11 years were obese in 1980, compared to nearly 18% in 2010. Because of the rising levels of obesity, the current generation of children may have a shorter life span than their parents for the first time in 200 years.

Childhood obesity has both immediate and long-term effects on health. The initial problems are usually psychological. Obese children often experience discrimination, leading to low self-esteem and depression. Their physical health also suffers. They are more likely to be at risk of cardiovascular disease from high cholesterol and high blood pressure. They may also develop pre-diabetes or diabetes type II. In the long-term, obese children tend to become obese adults, putting them at risk of premature death from stroke, heart disease, or cancer.

There are many factors that lead to childhood obesity and they often act in combination. A major risk factor, especially for younger children, is having at least one obese parent. The challenge lies in unravelling the complex links between the genetic and environmental factors that are likely to be involved.

**Why Was This Study Done?:**

Several studies have shown that a child's weight is influenced by his/her mother's weight before pregnancy and her weight gain during pregnancy. An obese mother, or a mother who puts on more pregnancy weight than average, is more likely to have an obese child.

One explanation for the effects of pregnancy weight gain is that the mother's overeating directly affects the baby's development. It may change the baby's brain and metabolism in such a way as to increase the child's long-term risk of obesity. Animal studies have confirmed that the offspring of overfed rats show these kinds of physiological changes. However, another possible explanation is that mother and baby share a similar genetic make-up and environment so that a child becomes obese from inheriting genetic risk factors, and growing up in a household where being overweight is the norm.

The studies in humans that have been carried out to date have not been able to distinguish between these explanations. Some have given conflicting results. The aim of this study was therefore to look for evidence of links between pregnancy weight gain and children's weight, using an approach that would separate the impact of genetic and environmental factors from a direct effect on the developing baby.

**What Did the Researchers Do and Find?:**

The researchers examined data from the population of the US state of Arkansas recorded between 2003 and 2011. They looked at the health records of over 42,000 women who had given birth to more than one child during this period. This gave them information about how much weight the women had gained during each of their pregnancies. The researchers also looked at the school records of the children, over 91,000 in total, which included the children's body mass index (BMI, which factors in both height and weight). They analyzed the data to see if there was a link between the mothers' pregnancy weight gain and the child's BMI at around 12 years of age. Most importantly, they looked at these links within families, comparing children born to the same mother. The rationale for this approach was that these children would share a similar genetic make-up and would have grown up in similar environments. By taking genetics and environment into account in this manner, any remaining evidence of an impact of pregnancy weight gain on the children's BMI would have to be explained by other factors.

The results showed that the amount of weight each mother gained in pregnancy predicted her children's BMI and the likelihood of her children being overweight or obese. For every additional kg the mother gained during pregnancy, the children's BMI increased by 0.022. The children of mothers who put on the most weight had a BMI that was on average 0.43 higher than the children whose mothers had put on the least weight.

The study leaves some questions unanswered, including whether the mother's weight before pregnancy makes a difference to their children's BMI. The researchers were not able to obtain these measurements, nor the weight of the fathers. There may have also been other factors that weren't measured that might explain the links that were found.

**What Do These Findings Mean?:**

This study shows that mothers who gain excessive weight during pregnancy increase the risk of their child becoming obese. This appears to be partly due to a direct effect on the developing baby.

These results represent a significant public health concern, even though the impact on an individual basis is relatively small. They could contribute to several hundred thousand cases of childhood obesity worldwide. Importantly, they also suggest that some cases could be prevented by measures to limit excessive weight gain during pregnancy. Such an approach could prove effective, as most mothers will not want to damage their child's health, and might therefore be highly motivated to change their behavior. However, because inadequate weight gain during pregnancy can also adversely affect the developing fetus, it will be essential for women to receive clear information about what constitutes optimal weight gain during pregnancy.

**Additional Information:**

Please access these websites via the online version of this summary at http://dx.doi.org/10.1371/journal.pmed.1001521.

- The US Centers for Disease Control and Prevention provide Childhood Obesity Facts
- The UK National Health Service article “How much weight will I put on during my pregnancy?” provides information on pregnancy and weight gain and links to related resources

## Introduction

The adverse effects of undernutrition during pregnancy on the long-term health of the offspring have been extensively studied [1],[2]. With onset of the epidemic of obesity in children, much attention has been focused on the consequences of maternal overnutrition, including high prepregnancy body mass index (BMI) and pregnancy weight gain [3]–[8]. Overnutrition during pregnancy has been hypothesized to alter fetal growth, body composition, metabolism, hormonal responses, and brain pathways regulating body weight or epigenetic patterns in ways that would increase long-term risk for obesity and related diseases.

Translational research provides evidence for this hypothesis [9]–[12]. In one study, offspring of female rats that were overfed before and during pregnancy gained more weight than offspring whose mothers were not overfed, even though both groups of progeny had the same genetic background [10]. Other animal studies reported that offspring of overfed mothers have increased expression of appetite stimulating neuropeptides, lower physical activity level, and changes in skeletal muscle structure and function [9],[11],[12].

Human studies have found associations between high prepregnancy BMI or pregnancy weight gain and body weight in the offspring [13]–[27]. However, the possibility of confounding must be carefully considered in these observational analyses of unrelated mother-child pairs. The child of an obese mother (or one who has gained excessive weight during her pregnancy) may also be obese, not because of the intrauterine effects of overnutrition, but rather because of shared obesity-promoting genetic or environmental factors. Indeed, among 2,758 families in the Collaborative Perinatal Project, the associations of prepregnancy BMI and pregnancy weight gain with child BMI z-score at age 4 years observed in conventional generalized equations became statistically nonsignificant in fixed effects models that account for shared familial influences [28]. Similarly, a study of 4,091 families found that the association between prepregnancy BMI and offspring adiposity at age 9 to 11 years became nonsignificant using the FTO (fat mass and obesity associated) genotype as an instrumental variable for maternal adiposity [29]. In addition, a sibling study of Swedish males born between 1973 and 1988 found no association between pregnancy weight gain and offspring BMI at age 18 years for the full cohort, though a positive association was observed among the subgroup of 21,146 offspring whose mothers had high prepregnancy BMI [30]. Moreover, most [31]–[36] but not all [37] analyses involving both parents demonstrate associations in BMI between father and offspring equal to, or greater than, between mother and offspring—findings that further discount a special influence of the intrauterine environment in this regard.

A recent observational study reported improved measures of adiposity among children born to 20 women after bariatric surgery, compared to their siblings born before surgery [38]. Here too, confounding by biological and behavioral factors cannot be excluded. Bariatric surgery may have produced changes in the mother (e.g., micro- or macronutrient malabsorption) or the household (e.g., greater awareness of the importance of weight control) that affected the offspring independent of maternal weight. Moreover, the children born after maternal bariatric surgery were substantially younger at the time of measurement (and their mothers were substantially older at the time of these children's birth), producing another source of potential confounding. Thus, apart from the special case of diabetes during pregnancy [39] in which severe metabolic aberrations may occur, the role of maternal overnutrition in childhood obesity remains unproven. Therefore, we aimed to examine the independent effects of pregnancy weight gain in a large, contemporary, population-based cohort with follow-up to a mean age of 11.9 years in the offspring, using a within-family design to minimize confounding.

## Methods

### Ethics Statement

These analyses were conducted under an approved Institutional Review Board (IRB) protocol through the University of Arkansas for Medical Sciences. The IRB at Princeton University also provided approval for this study. HLR had full access to, and takes responsibility for the integrity of, all primary data in the study. All authors vouch for the accuracy and completeness of the data analyses.

### Study Design

The purpose of this study was to determine whether the relationship between pregnancy weight gain and offspring weight previously observed at birth [40] persists into mid-childhood, using a similar, within-family analytic approach. We utilized Vital Statistics Natality records covering all live births in Arkansas from January 1, 1989 to December 31, 2005 and data on children's BMI mandated to be collected in all public schools from August 18, 2003 to June 2, 2011. Data for the current study were provided through a data use agreement with the Arkansas Center for Health Improvement (ACHI). ACHI is a legislatively governed entity authorized to collect and integrate statewide administrative datasets from the Departments of Health and Education, among others, through the use of strict data use agreements that protect the confidentiality and privacy of individual information contained in these records. ACHI conducted all data matching and de-identification of datasets prior to statistical analysis. Biological siblings (with the same mother) were matched using information contained in birth records based on maternal identifiers including name, date of birth, social security numbers, and sometimes addresses. Birth data were then linked to school BMI records using child-level identifiers including names, dates of birth, social security numbers, and sometimes addresses.

### Data Collection

Birth outcomes and maternal characteristics were obtained from Vital Statistics Natality records, which are based on the Certificate of Live Birth, a legal and medical record required for all births. (A summary of the data files is available from the US National Center for Health Statistics: http://www.cdc.gov/nchs/data_access/Vitalstatsonline.htm.) The variables of interest included pregnancy weight gain, birth weight, diabetes during pregnancy, week of gestation at delivery, maternal age, maternal education, maternal marital status, maternal smoking, child gender, child parity, and year of birth. Data for birth weight obtained by this method are highly reliable [41]. Prepregnancy weight is obtained from mothers (who complete a worksheet at the hospital) and weight at delivery is obtained from a worksheet completed by hospital personnel using labor and delivery records. Examples of the worksheets are available on the Centers for Disease Control and Prevention (CDC) Vital Statistics web page: (www.cdc.gov/nchs/data/dvs/momskf_improv.pdf and www.cdc.gov/nchs/data/dvs/facwksBF04.pdf).

Weight gain was calculated as weight at delivery minus maternally reported pre-pregnancy weight. Until very recently, prepregnancy weight and height were not included on the Certificate of Live Birth. Because prepregnancy weight is based on maternal self-report, it may have lower reliability than weight at delivery. However, an exact concordance between certificate of live birth data and medical record data for pregnancy weight gain was found in 82.8% of a random sample in North Carolina [42]. Another study found concordances between data on prepregnancy weight from the Certificate of Live Birth records and from medical records of 82.0% in New York City and 99.0% in Vermont [43].

A legislative act in Arkansas (Arkansas Annotated Code 20-7-133-135, 2003) mandated collection of height and weight for all public school children beginning in kindergarten. From 2004 through 2007, the state collected data for all children every year; beginning in 2008, data were collected every other year (i.e., kindergarten, 2nd, 4th, 6th, 8th, and 10th grades). During the fall semester of each school year, a data file of all public school students' demographic information was used to pre-populate data entry screens for schools to enter height and weight. Trained school personnel or student-health professionals obtained one weight and two height measurements at each time, as described [44]. BMI was calculated as (weight in pounds/[height in inches]^2^)×703. Gender- and age-specific BMI percentiles were calculated according to CDC definitions [45]. Students were classified as overweight or obese if BMI was ≥85th percentile.

Our study involved merging two administrative datasets collected by government agencies in Arkansas: the school student BMI records and the Vital Statistics Natality Data. Table 1 summarizes the sequential merger and exclusion process to obtain the study cohort. Beginning with 2,688,625 child BMI records from the Arkansas Dept. of Education, we excluded records lacking valid date of birth, height, or weight. Of these 2,222,521 school records, we were able to merge 1,044,086 (47.0%) with Vital Statistics Natality records. We then excluded multiple births, gestational age <37 weeks or >42 weeks, maternal diabetes, birth weight <500 g or ≥7,000 g, missing data for pregnancy weight, and weight gain outliers. Next, we deleted multiple records for the same child, including only the last record available, with the aim of assessing BMI of the offspring as late as possible in childhood. (Restricting measurement of BMI to a narrow age window would have substantially reduced the number of sibling pairs available, thereby reducing sample size and statistical power.) To control flexibly for differences in age, we added dummy variables for each month of age. Moreover, the median age difference between siblings was zero at the time of measurement (see below). Finally, per study design, we excluded children without siblings in the database, yielding a cohort of 91,045 offspring and their 42,133 mothers.

**Table 1:** Cohort selection and exclusion criteria.

Step	*n* Observations at End of Step
1. Child BMI records from Arkansas Dept. of Education.	2,688,625
2. Exclude records with missing or invalid data: - missing date of birth, gender, height, or weight (34,009) - not measured due to absence (190,162) - not measured due to disability (6,300) - not measured because child refused (64,737) - not measured because parent refused (95,805) - not measured because child no longer present in school; inaccurate enrollment record (73,074) - not measured due to pregnancy (1,776) - two height measures not within one inch (241)	2,222,521
3. Merge with Arkansas Vital Statistics Natality Records for 1989–2005	1,044,086
4. Exclude records with potentially confounding or erroneous data: - Gestation <37 or >42 weeks (*n* = 109,832) - Maternal diabetes (*n* = 18,808) - Multiple birth indicated in birth record (*n* = 10,605) - Birth weight <500 g or ≥7,000 g (*n* = 312) - Pregnancy weight gain missing (*n* = 62,591) - Pregnancy weight gain ≤−4.5 kg or ≥45 kg (*n* = 1,471)	840,467
5. Exclude all but last observation per child (*n* = 596,506)	243,961
6. Exclude records for children with no sibling in the merged birth-BMI dataset (*n* = 152,654).	91,045

Table 2 presents descriptive statistics for the children in our sample compared to all children born in Arkansas between 1989 and 2005 who satisfied similar sample exclusion restrictions (e.g., singleton births with gestation 37–42 weeks, excluding those with extreme values for birth weight). The characteristics of both groups were similar in most ways, including weight gain during pregnancy, although the study cohort had a slightly larger proportion of African Americans and married women, and a slightly lower proportion of smokers.

**Table 2:** Comparison of the study cohort with all Arkansas births 1989–2005.

Characteristics	Included in Analysis^a^	All Births in Arkansas, 1989–2005^b^
**Mother characteristics at birth**		
Total (*n*)	41,133	388,527
Maternal weight gain (kg)	13.9 (5.9)	14.0 (6.1)
Maternal age (years)	24.6 (5.2)	24.8 (6.1)
Mother's education (years)	12.6 (2.2)	12.5 (2.3)
Mother smoker (%)	17.9	19.2
Mother married (%)	72.6	68.1
**Child characteristics at birth**		
Total (*n*)	91,045	528,374
Birth weight (g)	3,416.5 (463.9)	3,386.7 (481.3)
Gestation duration (wk)	39.3 (1.1)	39.3 (1.2)
Offspring sex (% male)	50.9	51.0
Parity	1.6 (0.7)	2.0 (1.4)
Race/ethnicity, by maternal report (%)		
Black	19.1	18.4
Hispanic	2.6	4.5
Asian	0.7	0.9
**Child characteristic at time of last BMI measurement**		
Age (months)	142.68 (39.81)	—
Overweight or obese (%)	39.4	—

Data are mean (SD) or %.

a See Table 1 for a flow chart showing exclusions from the sample.

b Excluding preterm (<37 weeks) or post term (>42 weeks) gestational age, multiple gestational number, maternal diabetes, and extremes in birth weight (<900 g or >7,000 g).

### Statistical Analysis

The primary hypothesis was that pregnancy weight gain predicts child BMI, independent of confounding factors. We aimed to minimize confounding in three ways, following the approached used in our prior study of pregnancy weight gain and birth weight [40]. First, we eliminated some sources of potential confounding through sample exclusion criteria, including preterm (<37 weeks) or post term (>42 weeks) gestational age, multiple gestational number, maternal diabetes, and extremes in birth weight that may represent data entry error (<500 g or ≥7,000 g). Second, we incorporated measured confounders in our statistical models. Third, we controlled for residual confounding by measured and unmeasured (e.g., shared genetic and environmental) covariates by comparing offspring born to the same mother. Although we lacked data on prepregnancy BMI and paternal BMI because these variables are not included in the birth records in Arkansas, our inability to consider these covariates would not have produced spurious associations between pregnancy weight gain and childhood BMI (see Discussion).

The dependent variables were child BMI (expressed in a linear fashion) and the odds ratio (OR) for being overweight or obese, both determined at the last available measurement. Our main model regresses the dependent variable on continuous maternal weight gain. We also estimated models that regressed the dependent variable on indicators for the following categories of pregnancy weight gain: <6 kg, ≥12–18 kg, and >18 kg (using ≥6–12 kg as the reference category), an analytic approach that does not constrain the effects of weight gain to be linear. Covariates included in our models were sex of child, maternal marital status, an indicator for maternal smoking during pregnancy, child parity (indicators for parity of 1, 2, 3, 4 or more), the mother's age at the birth (<20, 20–24, 25–29, 30–34, ≥35), indicators for each month of child's age, indicators for each week of gestational age, and indicators for each year of birth (taking into account potential secular trends in childhood BMI unrelated to pregnancy weight gain). Where data on smoking and marital status were missing, we created a category for “missing” in addition to categories for other values, rather than deleting these observations from the dataset (*n* = 173 for smoking and *n* = 464 for marital status).

The models of child BMI as a continuous variable include maternal fixed effects and were estimated using the XTREG command in the STATA statistical program (release 11). In this way, we control for any maternal covariate that does not change with time (e.g., maternal height and weight before the first observed pregnancy, although neither was recorded in our database) [46]. XTREG “absorbs” the fixed effects by calculating the mean of the variable for each family and then subtracting the mean for the family from each individual observation. This procedure is mathematically identical, and produces identical estimates, to adding a separate indicator variable for each family, or (in the case of two-child families) to taking the difference between the two siblings. When there are more than two children per family (as is sometimes the case here), our procedure is more efficient than taking differences between adjacent siblings. We estimated standard errors that are robust to heteroskedasticity (i.e., subpopulations with different error variances) using the vce(robust) command in STATA [47]. For the binary outcome of overweight/obese or not, Fixed Effects Logit models were estimated using CLOGIT in STATA [48]. Further information about this statistical procedure is provided in Text S1.

To examine for effect mediation, we added birth weight of the child to the model involving BMI in two ways: as a continuous variable (in grams) and as categories, allowing for non-linear effects. Both models produced qualitatively similar results, and therefore only the linear model is presented. All data are presented as means and standard deviations (SDs) for maternal cohort characteristics or 95% confidence intervals (CI) for outcome data.

## Results

**Figure 1:**
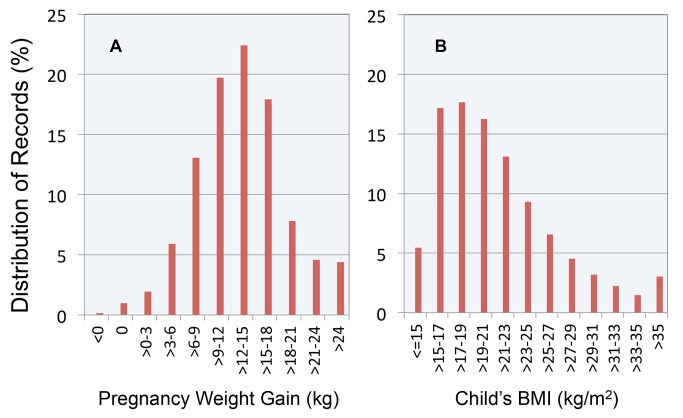
Distribution of maternal weight gain and child BMI in the study cohort. (A) Maternal weight gain; (B) child BMI.

As illustrated in Figure 1, the modal weight gain was 12 to 15 kg, accounting for 22.4% of pregnancies. The modal childhood BMI was between 17 and 19 kg/m^2^, with this category accounting for 17.7% of the sample. The distribution was right-skewed with significant numbers of children in the BMI categories 29–31 (3.2%), 31–33 (2.2%), 33–35 (1.5%), and >35 (3.0%).

Between successive pregnancies, the 10th percentile, 25th percentile, median, 75th percentile, and 90th percentile of changes in key covariates were: maternal pregnancy weight gain (−8.2, −4.1, 0.0, 3.6, 8.2 kg), birth weight (−595, −312, 0.0, 312, 595 g), child's last BMI (−7.5, −3.4, −0.1, 3.3, 7.4 kg/m^2^), and age of BMI measurement (−4.5, −2,4, 0.0, 2.5, 4.5 y). Thus, we found no evidence of systematic effects of birth order on any of these variables.

**Figure 2:**
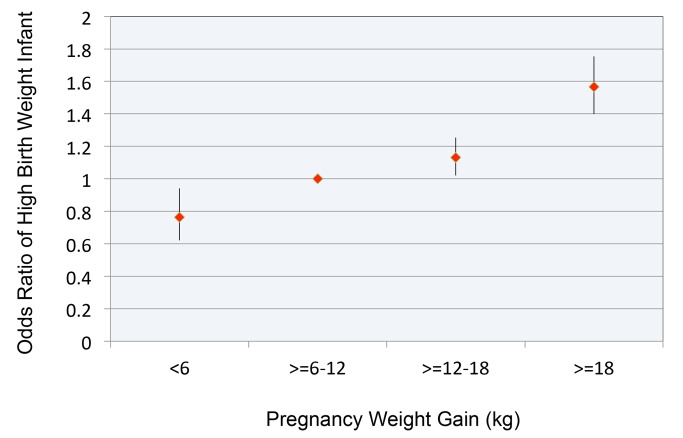
Relationship between pregnancy weight gain and odds ratio for high birth weight infant. Pregnancy weight gain expressed in kg. Reference range for pregnancy weight gain ≥6 to 12 kg. High birth weight defined as >4,000 g. Error bars are 95% confidence intervals.

Figure 2 replicates findings from our previous study [40], depicting the relationship between pregnancy weight gain and OR for birth weight >4,000 g. The OR of having a child with high birth weight, relative to the reference category, was 1.57 (CI 1.40–1.75, *p*<0.0001) for women who gained >18 kg.

In our primary analyses, each additional 1 kg of pregnancy weight gain as a continuous variable was associated with a 0.0220 (CI 0.0134–0.0306, *p*<0.0001) increase in childhood BMI and an OR of 1.007 (CI 1.003–1.012, *p* = 0.0008) for childhood overweight or obesity. (Tables S1 and S2 provide coefficient estimates for the models of BMI and OR for overweight/obese, respectively.) Adjustment for birth weight modestly attenuated (by approximately 35%) the association between pregnancy weight gain and child BMI. With this adjustment, childhood BMI increased by 0.0143 (CI 0.0057–0.0229, *p* = 0.0007) for every 1 kg of pregnancy weight gain.

**Figure 3:**
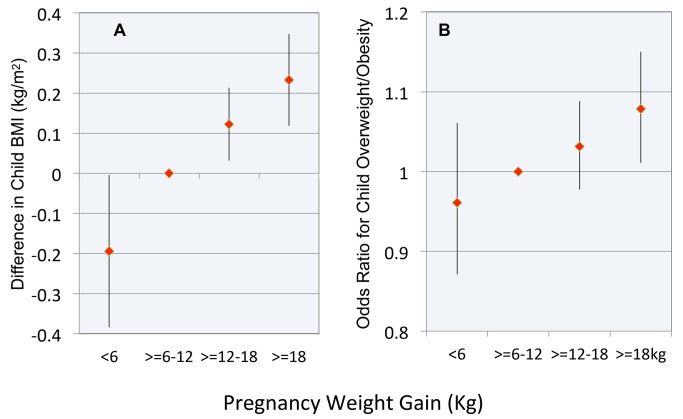
Relationship between pregnancy weight gain and body weight in childhood. (A) Difference in child BMI; (B) OR for child overweight or obesity. Reference range for pregnancy weight gain is ≥6 to 12 kg. Error bars are 95% confidence intervals.

The results of models involving pregnancy weight gain as a categorical variable demonstrated a nearly linear relationship with childhood BMI (Figure 3A) and the OR for childhood overweight or obesity (Figure 3B). The difference in childhood BMI associated with the lowest versus highest category of pregnancy weight gain was 0.43 kg/m^2^. A child whose mother gained >18 kg versus the reference range had a 1.08 OR (CI 1.01–1.15, *p* = 0.02) of overweight/obesity.

## Discussion

Using a population-based cohort contemporaneous with the obesity epidemic and a within-family design to minimize confounding, we found evidence for an independent association between pregnancy weight gain and body weight in childhood. We also observed an association between pregnancy weight gain and birth weight, replicating the finding from a prior study of births in New Jersey and Michigan [40]. However, birth weight mediated less than half of the association between pregnancy weight gain and child BMI. Because childhood body weight predicts adult body weight [49], our study suggests that overnutrition in pregnancy may program the fetus for an increased lifetime risk for obesity, though the magnitude of this effect may be small.

Excessive body weight in childhood is recognized to be complex in origin, and prevention of this highly prevalent medical problem will likely require attention to many modifiable factors. From this perspective, the magnitude of effects in our study, though small on an individual basis, could have important public health implications. Compared to the reference range, the 8% increase in risk among offspring of mothers with high pregnancy weight gain would account for several hundred thousand annual cases of pediatric overweight or obesity worldwide. In addition, for children whose mothers had high versus low pregnancy weight gain, the 0.43 kg/m^2^ increase in BMI could represent a significant component of the estimated 2 kg/m^2^ increase in mean childhood BMI in the US since the 1970s [50].

The main limitation of this study is our inability to consider the effect of prepregnancy BMI, because data on height and weight prior to conception were unavailable. Prepregnancy BMI could confound our findings if it were related to both pregnancy weight gain and childhood body weight. Indeed, prepregnancy BMI is positively associated with weight of the offspring later in life [16],[17],[19],[20],[23]–[26]. However, prepregnancy BMI is inversely associated with pregnancy weight gain [19]–[21],[24],[51]–[53]. Because heavier women gain less weight during pregnancy, on average, than normal weight women, our models would therefore tend to underestimate the magnitude of the associations involving pregnancy weight gain. Thus, our findings could not falsely arise from failure to consider prepregnancy BMI and the effect of including prepregnancy BMI into the statistical models, if any, would be to increase the magnitude of the associations. In any event, variations in prepregnancy BMI within individuals in our study would be substantially smaller than variations in conventional analyses involving comparisons between individuals, and the significance of weight change (typically weight gain) over time among women in our study would be further reduced by controlling for parity. Although adequately powered studies are needed to examine the joint effects of pregnancy weight gain and prepregnancy BMI, the former would probably have greater public health significance than the latter for several reasons. Weight management is easier over the short term (i.e., during pregnancy) than the long term (a woman's reproductive years), pregnant women tend to be especially motivated to pursue a healthful lifestyle out of concern for the well-being of their offspring, and many women do not know when they will become pregnant.

Several other study methodological issues warrant comment. The independent variable, pregnancy weight gain, is subject to reporting error that might vary by prepregnancy BMI, education, or levels of prenatal care, but any selective bias would be attenuated by our within-individual design. Random measurement error in any variable would tend to diminish the strengths of the associations, producing bias towards acceptance of the null hypothesis. In contrast, measurement of birth weight has high reliability [41], providing confidence in our estimates of effect mediation. We have no information about the Tanner stage in the BMI records, which are from an administrative school database. Conceivably, pregnancy weight gain could affect pubertal timing, and pubertal timing in turn could affect BMI. In addition, we have no information about paternal BMI. However, the increased genetic variation among siblings with different fathers would tend to decrease the precision, but not the accuracy, of our estimates. Furthermore, although the study cohort was derived from a state-wide registry, the generalizability of these findings to other populations with different baseline characteristics is not known.

Various biological or behavioral factors may mediate or modify our findings, warranting further mechanistically-oriented research. Pregnancy weight gain may influence childhood BMI, in part, through effects on infant body composition independent of birth weight or through programmed changes in body weight regulatory systems, as suggested by animal studies [9],[11],[12]. The effects of pregnancy weight gain on the offspring may also vary by weight status (prepregnancy BMI), diet quality, physical activity levels, genetic background, or other characteristics in the mother, and evidence of such effect modification could help identify populations at special risk of transgenerational obesity propagation.

In conclusion, this study suggests that high pregnancy weight gain increases body weight in childhood and that measures to limit pregnancy weight gain may help prevent obesity in the subsequent generation. However, in view of the observational nature of this study, additional research will be required to assess the relevance of these findings to obesity prevention on a public health basis.

## Supporting Information

Table S1 **Estimates from XTREG fixed effects model of child BMI on maternal weight gain (kg).**
(DOCX)

Table S2 **Fixed effects logit model for probability of child overweight or obese as a function of maternal weight gain during pregnancy (kg).**
(DOCX)

Text S1 **Notes on the estimation of sibling difference models.**
(DOCX)

## Abbreviations

BMI: body mass index
OR: odds ratio
