# Supplementary material for: Pregnancy Weight Gain and Childhood Body Weight: A Within-Family Comparison
Source: PLoS Med. 2013 Oct 1;10(10):e1001521. doi: 10.1371/journal.pmed.1001521 (PMC3794857; doi:10.1371/journal.pmed.1001521)
Supplement: Table S1 — Estimates from XTREG fixed effects model of child BMI on maternal weight gain (kg). (DOCX) [file pmed.1001521.s001.docx]

**Table S1: Estimates from XTREG Fixed Effects Model of Child BMI on Maternal Weight Gain (kg)**

|  | **Coef.** | **P>ItI** | **[95% Conf. Interval]** | |
| --- | --- | --- | --- | --- |
| Mother Weight Gain (kg) | 0.022 | 0.000 | 0.013 | 0.031 |
| Mother Age < 20 | 0.986 | 0.036 | 0.063 | 1.909 |
| Mother Age 20-24 | 0.884 | 0.058 | -0.030 | 1.798 |
| Mother Age 25-29 | 0.923 | 0.048 | 0.010 | 1.837 |
| Mother Age 30-34 | 1.000 | 0.034 | 0.074 | 1.925 |
| Mother Age 35+ | 1.165 | 0.018 | 0.202 | 2.129 |
| Mother Married | -0.003 | 0.976 | -0.171 | 0.165 |
| Married Missing | 2.051 | 0.014 | 0.419 | 3.683 |
| Mother Smoked | -0.161 | 0.068 | -0.334 | 0.012 |
| Smoking Missing | -0.369 | 0.332 | -1.115 | 0.377 |
| Child Male | -0.301 | 0.000 | -0.375 | -0.227 |
| Child 1st Born | 0.043 | 0.643 | -0.138 | 0.224 |
| Child 2nd Born | 0.029 | 0.671 | -0.106 | 0.165 |
| Child 4th or Higher Order | 0.083 | 0.610 | -0.236 | 0.401 |
| Gestation = 37 wks | 0.039 | 0.818 | -0.293 | 0.370 |
| Gestation = 38 wks | 0.031 | 0.847 | -0.281 | 0.342 |
| Gestation = 39 wks | 0.027 | 0.862 | -0.278 | 0.332 |
| Gestation = 40 wks | 0.033 | 0.828 | -0.267 | 0.334 |
| Gestation = 41 wks | 0.039 | 0.809 | -0.277 | 0.356 |
| Constant | 17.802 | 0.000 | 16.770 | 18.835 |

Notes: The model in Table S1 also included indicator variables for single month of child age. The omitted category of maternal age is “missing”. The omitted category of parity is 3^rd^ born. The omitted category of gestation is 42 weeks. The P-value refers to the probability that P>|t|.
