# Supplementary material for: Pregnancy Weight Gain and Childhood Body Weight: A Within-Family Comparison
Source: PLoS Med. 2013 Oct 1;10(10):e1001521. doi: 10.1371/journal.pmed.1001521 (PMC3794857; doi:10.1371/journal.pmed.1001521)
Supplement: Table S2 — Fixed effects logit model for probability of child overweight or obese as a function of maternal weight gain during pregnancy (kg). (DOCX) [file pmed.1001521.s002.docx]

**Table S2: Fixed Effects Logit Model for Probability of Child Overweight or Obese as a Function of Maternal Weight Gain During Pregnancy (kg)**

|  | **O.R.** | **P>ItI** | **[95% Conf. Interval]** | |
| --- | --- | --- | --- | --- |
| Mother Weight Gain (kg) | 1.007 | 0.002 | 1.003 | 1.012 |
| Mother Age < 20 | 1.410 | 0.129 | 0.904 | 2.198 |
| Mother Age 20-24 | 1.387 | 0.143 | 0.895 | 2.151 |
| Mother Age 25-29 | 1.418 | 0.120 | 0.913 | 2.201 |
| Mother Age 30-34 | 1.570 | 0.049 | 1.002 | 2.460 |
| Mother Age 35+ | 1.789 | 0.016 | 1.112 | 2.876 |
| Mother Married | 1.005 | 0.905 | 0.922 | 1.096 |
| Married Missing | 0.003 | 0.364 | 0.680 | 2.857 |
| Mother Smoked | 0.948 | 0.255 | 0.864 | 1.039 |
| Smoking Missing | 0.980 | 0.933 | 0.607 | 1.582 |
| Child Male | 1.066 | 0.003 | 1.022 | 1.112 |
| Child 1st Born | 1.054 | 0.661 | 0.834 | 1.331 |
| Child 2nd Born | 1.003 | 0.975 | 0.806 | 1.249 |
| Child 3rd Born | 0.972 | 0.793 | 0.783 | 1.205 |
| Gestation = 37 wks | 1.025 | 0.785 | 0.858 | 1.225 |
| Gestation = 38 wks | 1.021 | 0.811 | 0.864 | 1.205 |
| Gestation = 39 wks | 1.020 | 0.807 | 0.868 | 1.200 |
| Gestation = 40 wks | 0.981 | 0.808 | 0.836 | 1.149 |
| Gestation = 41 wks | 1.033 | 0.705 | 0.872 | 1.240 |

Notes: The model in Table S2 also included indicator variables for single month of child age. The omitted category of maternal age is “missing”. The omitted category of parity is 4^th^ or higher order. The omitted category of gestation is 42 weeks. The P-value refers to the probability that P>z.
