# Supplementary material for: Pregnancy Weight Gain and Childhood Body Weight: A Within-Family Comparison
Source: PLoS Med. 2013 Oct 1;10(10):e1001521. doi: 10.1371/journal.pmed.1001521 (PMC3794857; doi:10.1371/journal.pmed.1001521)
Supplement: Text S1 — Notes on the estimation of sibling difference models. (DOCX) [file pmed.1001521.s003.docx]

**Notes on the Estimation of Sibling Difference Models**

A model where siblings share certain characteristics (e.g. genetic endowments or home environment) can be represented as:

1. Y_it_ = a_i_ + Bx_it_ + e_it_,

Where the subscript ‘i’ indexes the family, while the subscript ‘t’ indexes the children within the family Y_it_, is the outcome of interest (e.g. child BMI), a_i_ represents the characteristics shared by both siblings that affect Y_it_, x_it_ are relevant covariates, and e_it_ is a random error term with mean zero. One way to think about this model is as one in which each family has a different initial level of child BMI. Equation (1) is also sometimes referred to as a multi-level model since there are two levels (the child and the family). This formulation appears on the second page of Gelman and Hill 2007, which addresses multilevel modeling (footnote 1).

Equation (1) can be estimated in several different ways. First, we could include >40,000 separate indicator variables for each family in the regression, though this approach is logistically cumbersome. Alternatively, we could obtain the differences between adjacent siblings. Suppose that there are 3 children per family such that t={1, 2, 3}. Then these differences would give us the following two observations for each family:

1. (y_i1_-y_i2_) = B(x_i1_-x_i2_) + (e_i1_-e_i2_) and (y_i2_-y_i3_) = B(x_i2_-x_i3_) + (e_i2_-e_i3_).

Clearly, differencing eliminates the family specific component, a_i_. This is the sense in which differencing controls for family characteristics.

A third way to deal with the a_i_ used here is to first take the mean of Equation (1) for each family. If we denote the mean of y as y*, etc. then we have:

1. Y_i_* = a_i_ + Bx_i_* + e_i_*

If we now subtract Equation (3) from Equation (1) we have:

1. (Y_it_ - Y_i_*)= B(x_it_ - x_i_*) + (e_it_ - e_i_*).

This method eliminates the family specific component a_i_ just as differencing eliminates the a_i_. If there are three children per family as in the example above, we can take (y_i1_-y_i_*), (y_i2_-y_i_*), (y_i3_-y_i_*), etc.

If there were exactly two children in each family, than differencing would be the same as subtracting out the family-specific mean. However, some families have more than two children, and in this case, differencing out the family-specific makes the most efficient use of all of the available information. One can appreciate this point by considering that the expression (y_i1_-y_i2_) ignores the available information about the third child, whereas the expression (y_i1_-y_i_*) makes use of the information about all the children in the family.

An additional question is why we estimate a fixed effects model rather than a random effects model. Most random effects models require the assumption that the random effect is uncorrelated with the error term. Also, the main advantage of the random effects model is that one can estimate a coefficient on variables that are fixed within groups (in this case within the family) but that is not of particular interest here.

We estimated fixed effects models in STATA using XTREG with robust standard errors

(generated using the vce option) which give accurate assessments of the sample-to-sample

variability of the parameter estimates even when the model is misspecified (e.g. if the

model is heteroskedastic or cluster-correlated). The robust variance within Stata is the

Huber/White/sandwich estimate of variance. We also looked at the correlation between the

family specific means and the error terms (=.037 for Table S1 below), which is a simple

diagnostic for whether the data is a good candidate for a random effects model. We found that

the fixed effects accounted for about half (.496 in Table S1 below) of the overall variance in

child BMI, suggesting that it is important to account for family specific effects.

Fixed-effects Logit models are estimated in STATA using CLOGIT. The fixed-effects logit model can be written as:

Pr(*yit* = 1 *|* **x***it*) = *F*(*αi* + **x***it****β***)

where *F* is the cumulative logistic distribution, *F*(*z*) = exp(*z*)/(1 + exp(*z*))

*i* = 1*,* 2*, . . . , n* denotes the independent units (called “groups” by clogit), and *t* = 1*,* 2*, . . . , Ti* denotes the observations for the *i*th unit (group).

When *Ti* is fixed, the maximum likelihood estimates for *αi* and ***β*** are inconsistent. Chamberlain (1980) showed that this problem could be solved by looking at the probability of

**y***i* = (*yi*1*, . . . , yiTi* ) conditional on the sum of the *yit* for each group. This conditional probability does not depend on *αi,* so they are not estimated.

A fixed-effects logit model has a format identical to a *k*1*i* : *k*2*i* matched case– control study discussed in Hosmer and Lemeshow (2000).

Estimates from the fixed effects logit model are shown in Table S2, below. One important issue with a fixed effects logit model is that groups of individuals who all have the same outcomes do not contribute to identification. In this case, families in which all of the siblings are overweight, or all of the siblings are not overweight, “drop out.” Hence, in Table S2 below there are 37,001 effective observations (i.e. children who had a sibling with a different outcome).

(For a more general discussion of longitudinal data modeling, see Hu et al. This article compares conditional likelihood with random effects approaches, including consideration of basic logit models and logit models with corrections to the standard errors to account for within-subject correlation.)

**References**

Allison PD (2009) Fixed effects regression models. Los Angeles: Sage Publications.

Angrist J, Pischke J-S (2009) “Mostly Harmless Econometrics: An Empiricist’s Companion,” Princeton University Press, Chapter 5.

Chamberlain G. (1980) Analysis of covariance with qualitative data. Review of Economic Studies 47: 225–238.

Gelman A, Hill J (2007) Data Analysis Using Regression and Multilevel/Hierarchical Models, Cambridge University Press, Chapter 1.

Hosmer DW, Lemeshow S (2000) Applied Logistic Regression. 2nd ed. New York: Wiley.

Hu FB, Goldberg J, Hedeker D, Flay BR, Pentz, MA (1998) Comparison of Population-Averaged and Subject-Specific Approaches for Analyzing Repeated Binary Outcomes, American Journal of Epidemiology 147 #7: 694-703.

Wooldridge J (2002) Econometric Analysis of Cross Section and Panel Data, MIT Press, Chapter 10.
